# Nip it in the bud: the role of vigilant leadership, knowledge sharing, and safety performance

**DOI:** 10.3389/fpubh.2025.1681592

**Published:** 2025-11-06

**Authors:** Qian Li, Song Lin Yang, Long Ye

**Affiliations:** School of Economics and Management, Beijing Jiaotong University, Beijing, China

**Keywords:** high-speed railway, vigilant leadership, safety performance, knowledge sharing, individual mindfulness, occupational health and safety

## Abstract

Maintaining vigilance is critical for High-speed railway (HSR) in a fickle environment of volatility, uncertainty, complexity, and ambiguity. A new vigilant leadership style was introduced into HSR literature, which directs employees to focus on, search for, and respond to potential future threats. This study constructs a research model to examine the effect of vigilant leadership on safety performance from a social information processing perspective, with individual mindfulness as boundary condition and knowledge sharing as a mediator. We adopted a time-lagged study with 961 samples and 137 workgroups collected from Chinese Railway Bureau over 3 months. The findings state that vigilant leadership is associated with employees’ safety performance by enhancing knowledge sharing. Employee’s mindfulness moderates the indirect effect of vigilant leadership on safety performance through knowledge sharing. The indirect effect is more positive when an employee’s mindfulness is high than when it is low. This research first introduces vigilant leadership to the Chinese HSR, which provides implications for raising the safety performance and long-term development of HSR organizations, and also benefits the HSR employees’ occupational health and safety.

## 1 Introduction

Maintaining safety and reliability is a perpetual theme for reliability-seeking organizations, striving to minimize or cut down the possibility of catastrophe close to zero (1, 2). Such organizations have been reported to include nuclear power plants (3), healthcare (4), airline/rail transportation (5), construction corporations (6), fire departments (7), and police departments (8). High-speed railways (HSR), as a typical one form an integral part of global transit, lauded for their rapidity, reliability, and eco-friendliness (9). While the occurrence of accidents in HSR organizations is uncommon, once such incidents happen, they can lead to severe injuries and fatalities (10–13). The National Rail Safety Action Plan Final Report (2005–2008) of the Federal Railway Administration (FRA) reported that human error accounted for approximately 38% of railway accidents, with a significant portion of 40% attributed to insufficient vigilance on the part of train drivers (14). Artificial intelligence is already widely used in HSR’s driving task to maintain safety, efficiency and reliability (15), but it is also the reason that causes a drop in HSR employees’ vigilance (10–13). Due to the nature of HSR, factors give impetus to improving driving safety and an employee’s vigilance is manifold. Among various factors, the leader is identified as a crucial component to be safe and reliable (16, 17). For instance, the Manchester Arena Bombing investigation report highlighted that the Security Service and Counter-Terrorism Policing unit conducted a preliminary inquiry into the bombers before the attack. However, due to the unit’s defective operational processes, they failed to take any subsequent preventive measures, ultimately causing the tragic incident (18). Cultivating leaders with a vigilance mindset can effectively mitigate the occurrence of such incidents (19). Thus, it is essential to perform research on a type of leadership style that focuses on improved vigilance and safety.

Zhao and Liu (20) identified the abovementioned leadership style as vigilant leadership. Compared with other leadership (e.g., safety-specific transformational leadership, transactional leadership), vigilant leadership incorporates alertness, curiosity, and a willingness to act on incomplete cues (21). Such leaders are attentive to subtle but significant signs of change, frequently challenge the existing success, encourage a value of never giving up in identifying and fixing minor issues quickly, and seek diverse perspectives (22, 23). Given the available literature and evidence (24), we have reasons to trust that vigilant leadership will be positively related to better safety outcomes in HSR organizations. The present research is prospective and forms part of an endeavor to test the relationships between vigilant leadership and safety performance by adopting the sample from Chinese HSR organizations.

Further, this study extends the interaction processes between the above relations to explain how and when vigilant leadership affects safety performance by introducing the notion of knowledge sharing. Sharing critical information and knowledge in HSR is critical in reducing experiential errors and preventing potential accidents (25). In the day-to-day operations of China’s HSR organization, vigilant leaders reiterate critical knowledge and underscore the safety production priorities on a weekly, monthly, and annual basis during the frequent study sessions. They encourage employees to cultivate a heightened sense of vigilance, which serves as an early warning for potential safety incidents and ensures the successful execution of frontline employees’ driving performance. The social information processing theory (SIP) (26) provided a theory supporting. It suggests that further processing knowledge and information can deepen employees’ understanding of the essence of the “Nip it in the bud” philosophy advocated by vigilant leadership, which leads to safer behaviors and better safety-related outcomes. The integrated safety model (27, 28) also indicates that distal situational-related factors (e.g., leadership) can influence safety behaviors through proximal factors (e.g., shared behavior). The unique demand for work experience and expertise determines the pivotal role of knowledge sharing in HSR (29). Consequently, we speculate that vigilant leadership impacts HSR employees’ safety performance through its effects on knowledge sharing via influencing employees’ further information processing.

Existing studies also demonstrate that individual traits significantly affect how leadership influences employees’ behavior (30, 31). We posit that as part of the information processing way, individual mindfulness serves as a beneficial moderator in the connection between vigilant leadership and knowledge sharing. HSR, as a mindful organization, advocates a mindfulness and vigilance culture (32, 33). Individual mindfulness pertains to how to dispose people process information in an alert, flexible approach (34, 35). It is about investigation and interpretation more grounded in capabilities for action (36). Hence, we infer that employee with high levels of mindfulness are usually committed to improving crisis awareness and deepening their understanding of vigilance culture in HSR organizations. They are more willing to share the code of action advocated by vigilant leaders as they better comprehend the importance of these actions in maintaining the safety and reliability. We therefore believe the underlying moderator mechanisms of individual mindfulness between vigilant leadership and knowledge sharing from an information processing perspective.

**Figure 1:**
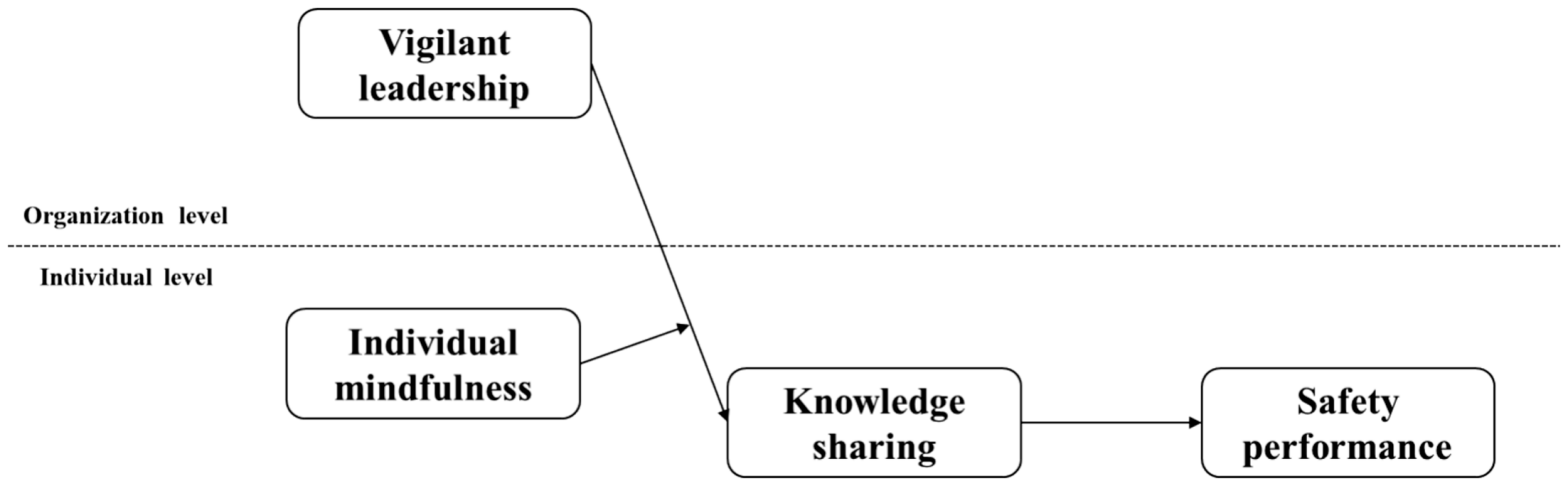
Proposed conceptual model.

In general, we build a conceptual model (as shown in Figure 1) to explore the direct and indirect effects of vigilant leadership on safety performance from an information processing perspective. This study makes three contributions: First, it not only expands research on the determinants of safety performance but also enriches empirical leadership studies in reliability-seeking organizations. Second, it extends the literature on leadership by examining how it influences safety performance by actively stimulating employees’ knowledge sharing from an information processing perspective. Third, it affirms the critical role of individual mindfulness in promoting knowledge sharing among HSR organizations, emphasizing the essential role of personal traits in processing leaders’ values.

## 2 Theoretical framework and hypotheses development

### 2.1 Vigilant leadership and safety performance

Vigilant leader was first proposed by Day and Schoemaker (19), who regarded it as a leader’s skill. Zhao et al. (37) defined it as leadership behaviors that guide employees to focus on, search for, and respond to potential threats. With respect to the latter, vigilant leadership is about alertness, guiding employees to prioritize potential problems and challenges instead of past achievements. Second, it centers on detection, encouraging employees to establish extensive external contacts to detect weak warning signals and possible adverse effects. Third, it is preventive, motivating employees to formulate preventive measures for potential challenges and threats and thereby reducing the negative impact of outbreaks. Generally speaking, vigilant leaders are both crisis-aware and forward-thinking, rather than focusing only on the current problem. They emphasize proactivity and prevention, direct employees’ attention to possible future threats and challenges (38). It is important to note that a leader who exhibits a vigilant style does not mean they are pessimistic about the future, but rather they put survival of the system and system boundaries first (37).

Safety performance for our purposes here seeks to achieve policies and regulations applicable to workplace safety (39, 40). The maintenance of safety performance within HSR is contingent upon the ongoing management of variations in both operational performance and interdepartmental interactions, as noted by Schulman (41). This necessitates a persistent focus on potential failures and anticipating unforeseen events that could arise in both immediate and distant future (42). Research indicates that leadership is one of the most vital determinants of safety performance in safety-oriented workplaces (16, 43), including transformational leadership, transactional leadership, and safety leadership (43–45). As these leadership styles are present-focused, they prioritize the resolution of immediate issues over potential future threats (46). There is a lack in the existing literature on future-oriented leadership styles that guide HSR to avoid future threats or failures (47). Leaders exhibiting a vigilant leadership style prioritize guiding their subordinates to address potential problems or challenges that lie ahead. In HSR organizations, attention to what was attempted, what could have happened, and why it did not happen is the most important source of maintaining organizational reliability and safety (48).

In the context of HSR organizations, a leader acts as a pivotal source of information within the workplace. According to social information processing (SIP) theory (26), their perspectives directly influence the attitudes and behaviors of employees (49). For HSR driving, drivers are typically required to work for approximately 4 consecutive hours within the confined space of the cabin (50). The nature of their duties, which inherently involve repetitive tasks, can lead to both physical fatigue and mental strain (51). These factors collectively heighten the risk of diminished vigilance among the drivers. Vigilant leaders instill a sense that safe driving requires careful navigation of obstacles or may fail at any time. They continually encourage employees to be proactive in examining various changes, looking for factors that pose a threat to operational reliability, and being prepared to guard against failures at any moment (37) because “nothing recedes like success” (48). These proactive approaches are often implemented through the use of walkie-talkies during driving or meetings (formally mandated) before and after driving. We inferred that under the influence of vigilant leadership, employees are more likely to form safety-oriented values and will be more inclined to adopt vigilance and rigor to avoid potential failures in achieving their goals, which is of great significance for raising individuals’ safety performance. Thus, we derive the following hypothesis:

*H1: Vigilant leadership is positively correlated with safety performance.*

### 2.2 Knowledge sharing as a mediator

The influence of specific information on individuals’ attitudes and behaviors requires a social interaction process to form or deepen employees’ understanding of values and cognition, thus further affecting their work attitudes and behaviors (26, 52, 53). Knowledge sharing is a suitable mechanism that helps employees gain a better comprehension of the vigilant leader’s perspective (54, 55). In HSR organizations, knowledge sharing refers to the transferability and interactivity of safety-related knowledge (e.g., veteran employees’ accident handling experience) among leaders and employees around a specific accident topic (56, 57).

Leaders are crucial in the knowledge sharing process (58, 59) because “words and deeds exhibited by leaders appreciate frontline employees’ contributions,” creating conditions for higher-quality information exchange (60). As the sender of early indicators of failures and unexpected events, leaders with a vigilance mindset convey the importance of “better safe than sorry” through teaching by example, thereby affecting employees’ values and further promoting knowledge-sharing behavior between and among employees. For example, in HSR organizations, vigilant leaders will facilitate frequent educational and training sessions aimed at anticipating and addressing potential issues or precursors to problems before arise, prompting subordinates to proactively implement preventive strategies. Additionally, incidents and accidents that have occurred are analyzed to enhance employees’ safety consciousness. Concurrently, employees who have experienced accidents are given cautions and undergo retraining, with a focus on reestablishing their vigilance. Employees are prompted to recognize the essential of maintaining vigilance. They embrace the concerns of the vigilant leader, integrating these into their code of conduct. This awareness is then conveyed to their colleagues, with understanding that disseminating this vital knowledge is crucial for everyone’s safety driving.

Meanwhile, a vigilant leader nurtures a culture of vigilance and discovery among employees, urging them to pay attention to subtle but critical change signs and spreading the acquisition of vital experience (61), thereby helping employees to redesign their understanding of crisis and risk (62). This in turn helps create a stable foundation for detecting weak signals and unexpected events sooner while simultaneously aiding diverse understandings and faster adaptation (63, 64). It also enables employees to understand the value of transmitting reliability-related safety knowledge, which contributes to knowledge sharing consciously and voluntarily (65).

Vigilant leaders also influence the willingness of employees to share knowledge by projecting leadership. Vigilant leaders can describe a blueprint for the organization and establish a shared vision that makes employees recognize the useful function of constant vigilance, resilience, and expertise (21, 66). They assist employees in achieving reliability through support, guidance, and motivation. This transmission of vision and beliefs seeks to highlight the paramount role of leaders in addressing potential future threats in their work, deepening or shaping employees’ understanding of the value and identity of achieving safety and reliability value within HROs like HSR (65). Moreover, sharing safety knowledge is crucial for HSR employees’ safety performance (67, 68). Knowledge sharing strengthens employees’ identification with the HSR organization’s common goal, improves the quality of communication among employees, and motivates them to absorb and learn from the perspectives of others inside the organization, to master more skills and gain indirect experiences to raise safety performance (69, 70). At the same time, a higher level of knowledge sharing also encourages individuals to effectively help colleagues solve problems and coordinate challenging tasks (71, 72), ensuring high reliability. In sum, the following hypothesis is proposed:

*H2: Knowledge sharing plays a mediating role between vigilant leadership and safety performance.*

### 2.3 Individual mindfulness as a moderator

Mindfulness embodies a versatile mental state centered on the present, characterized by an alertness to novelty and a keen awareness of one’s surroundings (73, 74), which we treated as a trait in the current study. It represents an indispensable ability for HSR employees, enabling them to monitor and manage moment-to-moment experiences with curiosity, openness, and acceptance (34). More precisely, individual mindfulness stresses (1) meticulous attention to detail; (2) active engagement with the present moment; (3) a flexible state of mind; and (4) a willingness to embrace diverse and unfolding realities (75). Employees who possess mindfulness are inclined to detect/dissect intricate interconnections (76) and detect/anticipate weak signals and possible threats to reliability (77). In HSR organizations, employees are required to pay constant attention to the train’s internal operational condition; in this process, the employees also needs to remain vigilant for the external environment, including radio communications, vehicle intersections, and the prevailing weather and regional conditions surrounding the train (10–13). Employees with a mindfulness mindset demonstrate enhanced proficiency in detecting and responding to nuanced and emerging cues (78). They are adept at identifying and analyzing intricate, often subtle, interconnections (79). This trait fosters employees’ better comprehension of the perspectives maintained by vigilant leaders, thereby increasing the likelihood of generating more robust knowledge sharing behaviors. More specifically, when employees observe their actions or value orientation align with the value propositions championed by vigilant leaders, they will experience a sense of validation. This leads to an increased likelihood of actively sharing what they consider to be accurate behaviors and knowledge with peers, effectively boosting knowledge-sharing behaviors.

Employees with low-level mindfulness are unable to recognize emerging and weak signals, delay, or refuse to take action when faced with a highly variable and uncertain task environment. They are relatively insensitive to the detail and vigilance work atmosphere created by vigilant leadership, and less likely to process information conveyed by the leader. We infer that individuals’ elevated level of mindfulness will enhance the positive influence of vigilant leadership exerts on knowledge sharing. Conversely, a lower degree of mindfulness tends to diminish the beneficial effects of vigilant leadership on the process of knowledge sharing. Consequently, we suggest the following proposition:

*H3: Individual mindfulness positively moderates the relationship between vigilant leadership and knowledge sharing.*

The proposed assumptions regarding the moderating effects and the indirect relationship suggest a moderated mediation model. This model implies that the total indirect effect of vigilant leadership on safety performance, mediated by knowledge sharing, is moderated by individual mindfulness. When leaders exhibit a high level of vigilance, they consistently foster vigilance awareness among their subordinates and encourage proactive approaches to handling crises. Consequently, employees are more inclined to engage in knowledge sharing activities. Meanwhile, employees with greater mindfulness are more apt to follow through on their leaders’ intentions, leading to increased knowledge sharing and ultimately improving safety performance. Therefore, we propose the following hypothesis:

*H4: Individual mindfulness moderates the indirect effect of vigilant leadership on safety performance through knowledge sharing, such that the indirect effect is more positive when individual mindfulness is high than when it is low.*

## 3 Method

### 3.1 Sample and procedure

We chose China’s HSR as our research object for the following reasons: First, according to the National Railway Administration of China reports that as of the end of 2024, China’s HSR network spans 48,000 kilometers. It accounts for more than 70% of the total mileage of HSRs worldwide, which makes China a valuable context for studying HSR. Second, the availability of the data. This research relies on the National Natural Science Foundation of China and the National Key Research and Development Program of the Ministry of Transport of China, and we have the opportunity to conduct a formal survey to obtain data regarding the actual conditions of HSR employees in China. Third, the importance of leadership with a sense of vigilance in reliability-seeking organizations like HSR (80). In the Chinese HSR, leaders play a significant role in practical work. For instance, the leaders emphasize the key points at the morning meeting every day, nipping any potential accidents in the bud. Every month, organized and carried out training sessions, helping employees to memorize the handling methods of typical accident cases.

Generally speaking, our research data was mainly collected from the Railway Bureau in China (Shanghai, Nanjing, Chongqing and Guangzhou). We invited 1,055 railway employees from 153 workgroups to participate in this research. The purpose of our research was clearly explained, and it was emphasized that participants were free to decline involvement and their answers would be confidential. All procedures were examined and approved by the Ethics Committee of Economics and Management in Beijing Jiaotong University (protocol code ECSEM2023031001), which was conducted in accordance with the local legislation and institutional requirements.

At Time 1(T1), 1,055 employees rated their demographic information (age, education, gender, marital status, working years), and the individual mindfulness, their perception of vigilant leadership. 153 supervisors rated their age, education, and working years. T2 was conducted with a three-week time lag. We asked these 1,055 employees to provide data on knowledge sharing. A three-week later (T3), the same 1,055 employees’ safety performance data over the past month was obtained from the Railway Bureau, and 1,032 matched questionnaires were collected. After excluding 71 invalid questionnaires (omissions, incorrect completions or less than 4 completions from a single workgroup), a total of 961 fully completed questionnaires from 137 workgroups were obtained and subjected to further analysis. We asked them to mention their job number as identification, which was used for matching data from T1, T2, and T3 (81).

Upon executing a confidentiality agreement, we gathered the performance evaluation scores for each participant to lay a foundation for rating their safety performance. The Chinese HSR organization has a sophisticated safety assessment and rating system that can accurately measure safety violations and apply appropriate point deductions. The assessment covers multiple key operational procedures, such as the pantograph raising procedures, the use of traction modes, and pointing and calling actions, each with clearly defined score allocations. The final score serves as an indicator of the differences in employees’ safety performance. Based on this continuous indicator, the present study utilized the performance scores collected at T3 as the measure of safety performance, and subsequently conducted hierarchical linear modeling (HLM) and multilevel structural equation modeling (MSEM) analyses based on this data (82). A negative scoring method was employed, where lower scores mean poorer safety performance.

Among these data, for employees, 99.8% were male. 62.3% had a bachelor’s degree, 37.5% were below a bachelor’s degree. Further, 54.3% are unmarried, and 45.7% got married. In addition, 44.2% had 6 ~ 8 years of working experience, 22.5% had 3 ~ 5 work years, and 25.9% had less than 3 years. For supervisors, 100% were male. 31.4% were below a bachelor’s degree, 39.2% had a bachelor’s degree, and 29.4% had a postgraduate degree; 24.8% less than 2 work years, 11.1% had 3 ~ 5 work years, 33.3% had 6 ~ 8 work years, 21.6% had 8 ~ 11 work years, and 9.2% had more than 12 work years.

### 3.2 Measures

Scales were translated into Chinese following the translation and back-translation procedure of Brislin (83). All measures were scored on a 5-point Likert-type scale. These scales are widely accepted by scholars.

#### 3.2.1 Vigilant leadership

The scale developed by Zhao et al. (37) was adopted. It has a total of nine items. Examples included “Encourage employees to draw lessons from security incidents or unsafe incidents encountered by other enterprises.” (Cronbach’sα = 0.955).

#### 3.2.2 Knowledge sharing

We assessed KS by adapting a four-item scale (84). This scale contains two subdimensions: “donating” (five items) and “collecting” (four items). We chose “collecting” as the measurement tool. Sample items included “Colleagues within my department tell me what they know when I ask them about it.” (Cronbach’sα = 0.872).

#### 3.2.3 Individual mindfulness

We modified the 15-item scale by Brown and Ryan (85). Sample items included “I tend to walk quickly to get where I’m going without paying attention to what I experience along the way.” (Cronbach’sα = 0.965).

#### 3.2.4 Control variables

Based on prior studies on relevant issues, the current study selected gender, age, educational background, marital status, and working years as control variables when testing the proposed hypotheses (86).

### 3.3 Data analysis

Since this study involved employees nested in 137 groups (with an average group size of 7.01, ranging from 3 to 12), we report the design effect (DEFF). Based on the ICC (1) of vigilant leadership, the Kish-corrected DEFF is approximately 5.03. This implies that the effective sample size is about 961 ÷ 5.03 ≈ 191. Compared to the scenario where individuals are completely independent, the variance is approximately five times greater. Therefore, this study employs a multilevel modeling approach and introduces cluster-robust standard errors in robustness checks to ensure the reliability of the estimation results.

Meanwhile, given that the sample size declines from 1,055 → 1,032 → 961, with 71 cases excluded, we provide an attrition analysis. The results of Little’s MCAR test ( $\chi^{2}\;$ = 12.37, df = 14, *p* = 0.57) demonstrated that no significant differences in T1 covariates (age, gender, tenure, education, marital status) between the retained and attrition groups, suggesting that sample attrition did not lead to systematic bias. To ensure the rigor of the time-lagged analysis, we only retained samples with complete data across three time points and did not employ imputation methods, so as to avoid potential biases caused by manually completing the data.

Generally speaking, data analysis was conducted using SPSS 26.0, including PROCESS 4.2, AMOS 24.0, R Language, and MPLUS 8.0. Initially, SPSS was used for descriptive statistics and correlation analysis. Then, confirmatory factor analysis (CFA) was conducted using AMOS to gain evidence of the congruence between measurement factors and scale items as per the structure of our research model and to evaluate the discriminant validity of each measure (vigilant leadership, knowledge sharing, individual mindfulness, and safety performance). Multilevel confirmatory factor analyses (MCFA) were carried out to further assess the distinctiveness of the model constructs (87). Next, the common method bias test was also performed by SPSS to ensure the validity of the self-reported survey. Finally, the multilevel data modeling methods of HLM and MSEM were applied to test our research hypotheses.

The direct effects hypothesis (Hypothesis 1), the mediation hypothesis (Hypothesis 2), and the moderation hypothesis (Hypothesis 3) were estimated using HLM through the R Language. Additionally, a simple slope analysis was performed to examine the nature of the moderating effect (88). The moderated mediation hypotheses (Hypothesis 4) were examined using MPLUS (89).

### 3.4 Aggregation

The within-group agreement (*r_wg_*) and intraclass correlations (ICC (1) and ICC (2)) were used to demonstrate the rationality of the data aggregations (90). Specifically, *r_wg_* compares the variance of low-level variables with that of random distributions to determine the rationality of adding low-level variables to high-level variables. ICC is a comparison of the variance within-group and the average variance between-group represented as ICC (1) and ICC (2), respectively. Bliese (91) reported that if *r_wg_*, ICC (1) and ICC (2) are greater than 0.70, 0.05 and 0.60, respectively, then individual level variables can be aggregated to the team level. The median *r_wg_* value for Vigilant leadership was 0.93 > 0.7. The value of ICC (1) was 0.59 > 0.05, and ICC (2) was 0.91 > 0.6. The results prove that the aggregation is rational.

We also adopted different centering decisions. Using group-mean centering for individual-level variables (such as individual mindfulness, knowledge sharing) to distinguish within-group effects from between-group effects; Using grand-mean centering for group-level variables (such as aggregated vigilant leadership) to facilitate cross-group comparison and interpretation (91).

## 4 Results

### 4.1 Descriptive statistics and correlations

Table 1 presents the means, standard deviations, and correlations among the variables in the study. The vigilant leadership on the individual level is perceived by employees, and vigilant leadership on the leader level is aggregation. The correlational analysis results indicated that vigilant leadership is positively related to knowledge sharing (r = 0.416, *p* < 0.01), safety performance (r = 0.206, p < 0.01); knowledge sharing is also positively associated with safety performance (r = 0.541, p < 0.01). The initial results offer some evidence in support of our proposed correlations.

**Table 1:** Descriptive statistics and correlations results.

Variable	M	SD	1	2	3	4	5	6	7	8	9
Individual											
1. Gender	1.998	0.043	——								
2. Working years	2.342	0.968	−0.007	——							
3. Education	3.615	0.524	0.01	0.469**	——						
4. Marriage	1.457	0.498	−0.004	0.571**	0.334**	——					
5. Age	27.213	3.043	0.01	0.837**	0.466**	0.603**	——				
6. Knowledge sharing	3.701	0.98	0.02	0.007	0.069*	0.042	0.001	——			
7. Safety performance	−30.168	22.433	−0.053	0.004	0.025	−0.030	−0.023	0.538**	——		
8. Individual mindfulness	3.211	1.057	−0.059	−0.052	−0.026	0.024	−0.016	−0.110**	−0.099	——	
9. Vigilant leadership	3.39	1.164	−0.008	−0.012	0.026	−0.015	−0.045	0.416**	0.300**	−0.136**	
The range of factor loadings	——	——	——	——	——	——	——	0.71–0.84	0.81–0.94	0.75–0.92	0.85–0.94
AVE	——	——	——	——	——	——	——	0.624	0.612	0.554	0.544
CR	——	——	——	——	——	——	——	0.863	0.832	0.894	0.812
Leader											
1. Vigilant leadership	3.577	0.869	——								
2. Education	2.11	0.773	−0.056	——							
3. Age	2.394	1.583	0.093	0.007	——						
4. Working years	2.781	1.241	0.347**	−0.005	0.625**	——					

*p < 0.05; **p < 0.01.

### 4.2 Confirmatory factor analyses

Model fit was evaluated by calculating the chi-square statistic (92). We took into consideration the RMSEA (Root Mean Square Error of Approximation), CFI (Comparative Fit Index), TLI (Tucker-Lewis Index), and SRMR (Standardized Root-Mean-square Residuals) goodness of fit statistics. The explanation of these indexes as follows: RMSEA <0.08 = acceptable model (93); CFI > 0.90 = acceptable model, and >0.95 = excellent model (94); TLI > 0.90 = acceptable model, and >0.95 = excellent model (95); SRMR <0.08 = acceptable mode (96).

The findings of CFA (Table 2) demonstrate that the four-factor model provided a more reasonable and superior fit than the other models (χ^2^/df = 1.281 < 3, CFI = 0.931 > 0.9, TLI = 0.928 > 0.9, RMSEA = 0.031 < 0.08, SRMR = 0.035 < 0.08). These indexes indicated that the model fits the data well. Therefore, we believe that the research variables are properly distinguished. In addition, MCFA were performed to further validate the distinctiveness of the four-factor model, which is regarded as an effective hierarchical structure analysis method to be utilized in leadership research (97). The analysis results of the MCFA demonstrate again that the four-factor model superior fit than the other models (Table 2).

**Table 2:** Confirmatory factor analysis and multilevel confirmatory factor analysis results.

Models	χ^2^/Df	Df	CFI	TLI	RMSEA	SRMR
1. Four factors: Vigilant leadership, knowledge sharing, safety performance, individual mindfulness	1.281	372	0.931	0.928	0.031	0.035
2. Three factors: Knowledge sharing and safety performance combined	2.321	374	0.860	0.869	0.087	0.095
3. Two factors: Knowledge sharing, vigilant leadership, and safety performance combined	8.121	376	0.760	0.792	0.112	0.107
4. One factor: All factors combined	34.134	377	0.537	0.501	0.177	0.409
MCFA 4	1.182	584	0.936	0.936	0.026	W = 0.021, B = 0.029

We conducted Harman’s single-factor test to assess potential common method bias among the variables. The findings revealed that the variance explained by the largest factor accounted for 39.54%, which was under the empirical criterion of 40% (10–13). This suggests that the study did not suffer a serious problem of common methodological bias. Considering Harman’s single-factor test is a somewhat lenient method for assessing common method bias, we adopted a method incorporating latent factors to further examine the common method bias (98). Adding unmeasured latent factors resulted in a slight decline in model fit (Δχ^2^ = 10.21, ΔRMSEA<0.001, ΔCFI< 0.001, ΔTLI< 0.001), suggesting no significant impact of common method bias.

### 4.3 Hypotheses testing

#### 4.3.1 Direct effect testing

Hypothesis 1 proposed a positive association between vigilant leadership and safety performance. Our analysis yields a positive relation between vigilant leadership and safety performance as shown in Table 3 (β = 0.220, *p* < 0.001, Model 5), which means that vigilant leadership significantly enhances the safety performance of subordinates. Hypothesis 1 was supported.

**Table 3:** Hierarchical linear modeling analysis results.

Variable	Knowledge sharing			Safety performance		
	Model 1	Model 2	Model 3	Model 4	Model 5	Model 6
Intercept ($\gamma_{00})$	3.785*** [3.667, 3.903]	3.784*** [3.672, 3.895]	3.788*** [3.676, 3.900]	0.006 [−0.102, 0.115]	0.005 [−0.094, 0.105]	0.006 [−0.094, 0.106]
Level-1						
Gender	0.021 [−0.067, 0.109]	0.346 [−0.048, 0.740]	0.413* [0.033, 0.794]	0.076 [−0.436, 0.587]	0.099 [−0.408, 0.607]	−0.02 [−0.125, 0.085]
Age	0.007 [−0.021, 0.035]	0.01 [−0.018, 0.038]	0.012 [−0.015, 0.039]	0.006 [−0.031, 0.042]	0.01 [−0.026, 0.046]	0.007 [−0.028, 0.041]
Working years	−0.018 [−0.103, 0.067]	−0.025 [−0.109, 0.060]	−0.034 [−0.116, 0.047]	−0.019 [−0.128, 0.091]	−0.027 [−0.136, 0.082]	−0.02 [−0.508, 0.469]
Marriage	−0.061 [−0.176, 0.053]	−0.059 [−0.172, 0.055]	−0.062 [−0.171, 0.048]	−0.053 [−0.201, 0.094]	−0.049 [−0.195, 0.097]	−0.028 [−0.169, 0.112]
Education	0.048 [−0.054, 0.150]	0.039 [−0.062, 0.140]	0.041 [−0.056, 0.139]	−0.006 [−0.138, 0.125]	−0.018 [−0.149, 0.112]	−0.034 [−0.159, 0.092]
Knowledge sharing						0.352*** [0.267, 0.437]
Individual mindfulness ( $\gamma_{01})$			−0.161*** [−0.203, 0.120]			
Level-2						
Leader Age	−0.025 [−0.077, 0.027]	−0.02 [−0.071, 0.031]	−0.018 [−0.067, 0.031]	−0.019 [−0.081, 0.042]	−0.004 [−0.063, 0.056]	−0.003 [−0.061, 0.055]
Leader Working years	0.047 [−0.016, 0.110]	0.034 [−0.028, 0.096]	0.031 [−0.029, 0.091]	0.045 [−0.030, 0.120]	0.01 [−0.063, 0.084]	0.009 [−0.063, 0.080]
Leader Education	−0.017 [−0.084, 0.050]	−0.013 [−0.079, 0.053]	−0.013 [−0.077, 0.050]	−0.003 [−0.085, 0.080]	0.004 [−0.076, 0.085]	0.008 [−0.070, 0.086]
Vigilant leadership		0.158*** [0.099, 0.216]	0.129*** [0.072, 0.186]		0.220*** [0.151, 0.289]	0.188***
						[0.120, 0.255]
Interaction term						
Vigilant leadership* Individual mindfulness ( $\gamma_{11})$			0.053** [0.014, 0.092]			
Intraclass variance $\;\sigma^{2}$	0.419	0.371	0.378	0.303	0.24	0.253
Between-column variance $\tau_{00}$	0.418	0.412	0.383	0.695	0.684	0.632
ICC	0.5	0.473	0.496	0.303	0.259	0.286
Marginal *R^2^*	0.005	0.042	0.066	0.002	0.057	0.093
Conditional *R^2^*	0.503	0.496	0.53	0.305	0.302	0.353
*Omega^2^*	0.563	0.569	0.601	0.383	0.389	0.438

Marginal *R*^2^: fixed effects; Conditional *R*^2^: fixed + random effects; Omega^2^ = 1—proportion of unexplained variance; Standard error in parentheses; *p < 0.05, **p < 0.01, and ***p < 0.001. The values in “[]” are confidence intervals.

#### 4.3.2 Mediating effect testing

Hypothesis 2 proposed that the effect of vigilant leadership on safety performance was mediated through knowledge sharing. According to the analysis results (Table 3), model 2 shows a significant positive association between vigilant leadership and knowledge sharing (β = 0.158, *p* < 0.001). Model 5 indicates that knowledge sharing had a positive impact on safety performance (β = 0.352, *p* < 0.001). This suggests that knowledge sharing plays a key role in both pathways. The significance of the mediation effect is further confirmed by the outcomes of the Monte Carlo Test in Table 4. The estimate of within indirect effect for knowledge sharing was 0.104 (*p* = 0.0499, 95% confidence interval [0.003, 0.210]) and the between indirect effect was 0.399 (*p* < 0.001, 95% confidence interval [0.194, 0.616]). The findings indicate that vigilant leadership has a notable indirect influence on safety performance through knowledge sharing across hierarchical levels. The impact of both within and between indirect effects lends credence to the hypothesis of a cross-level mediating effect, thereby affirming the pivotal role of knowledge sharing in raising safety outcomes.

**Table 4:** Monte Carlo test results for cross-level mediation effect.

Effect types	Estimate	S.E.	*p* value	95% confidence interval	
				MCLL	MCUL
Within Indirect Effect	0.104	0.053	0.0499	0.003	0.21
Between Indirect Effect	0.399	0.107	0	0.194	0.616
Within Direct Effect	0.113	0.023	0	0.068	0.157
Between Direct Effect	0.083	0.026	0.002	0.031	0.135

#### 4.3.3 Moderating effect testing

HLM was also used to test the moderating effect of individual mindfulness on the relationship between vigilant leadership and knowledge sharing. The results are shown in Table 3. Table 3 stated that the interaction item of vigilant leadership with individual mindfulness (*γ* = 0.053, *p* < 0.001, Model 3) had a significant impact on knowledge sharing, providing support for hypothesis 3. This highlights that the enhancing impact of vigilant leadership on knowledge sharing is particularly pronounced when individual mindfulness levels are high, and conversely, the influence is notably diminished at lower levels of individual mindfulness. This further emphasizes the critical role of individual mindfulness as a cross-level moderator variable, reflecting the moderating effect of individual mindfulness on the relationship between vigilant leadership and knowledge sharing in different situations.

**Figure 2:**
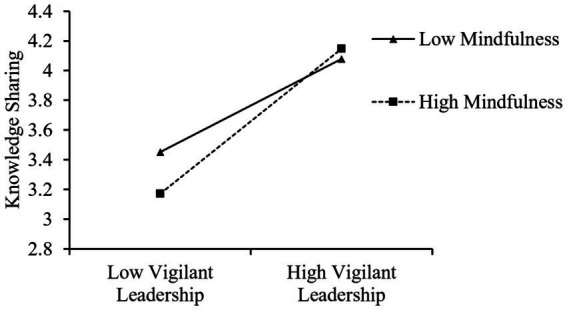
The moderating effect of individual mindfulness.

To better describe the moderating effect of individual mindfulness, we did a further test providing a figure of the simple slope moderating effect (±1SD). As depicted in Figure 2, vigilant leadership had a more significant positive impact on knowledge sharing when individual mindfulness was high.

Finally, taken together, Hypothesis 4 proposed a moderated mediation in which the interaction between vigilant leadership and safety performance through knowledge sharing is moderated by individual mindfulness (IM). To examine the integrative moderated mediation model, MSEM was applied, which was combined with Monte Carlo bootstrapping analysis (5,000 resamples). The 95% confidence intervals for the conditional indirect effects are shown in Table 5. As we can see in Table 5, the indirect effect of vigilant leadership on safety performance via knowledge sharing was stronger when individual mindfulness was high (indirect effect when IM was high = 0.411, CI [0.348, 0.474]; indirect effect when IM was low = 0.256, CI [0.215, 0.297]). The difference between the indirect effects at high and low levels of IM was 0.155, with a 95% CI of [0.084, 0.226]. These results support Hypothesis 4.

**Table 5:** Moderated mediation results.

Leve	B	S.E.	[95% CI]
Low individual mindfulness	0.256	0.021	[0.215, 0.297]
High individual mindfulness	0.411	0.032	[0.348, 0.474]
Difference	0.155	0.036	[0.084, 0.226]

Besides, we conducted a simulation analysis of statistical power by using the R package simr. The results indicate that the statistical power for main effects (vigilant leadership → safety performance, vigilant leadership → knowledge sharing, and knowledge sharing → safety performance) all exceed 0.80, demonstrating that the estimates are relatively robust (99). However, the interaction effect (vigilant leadership × individual mindfulness → knowledge sharing) is only 64%, which indicates that limited statistical power under the current sample size.

**Figure 3:**
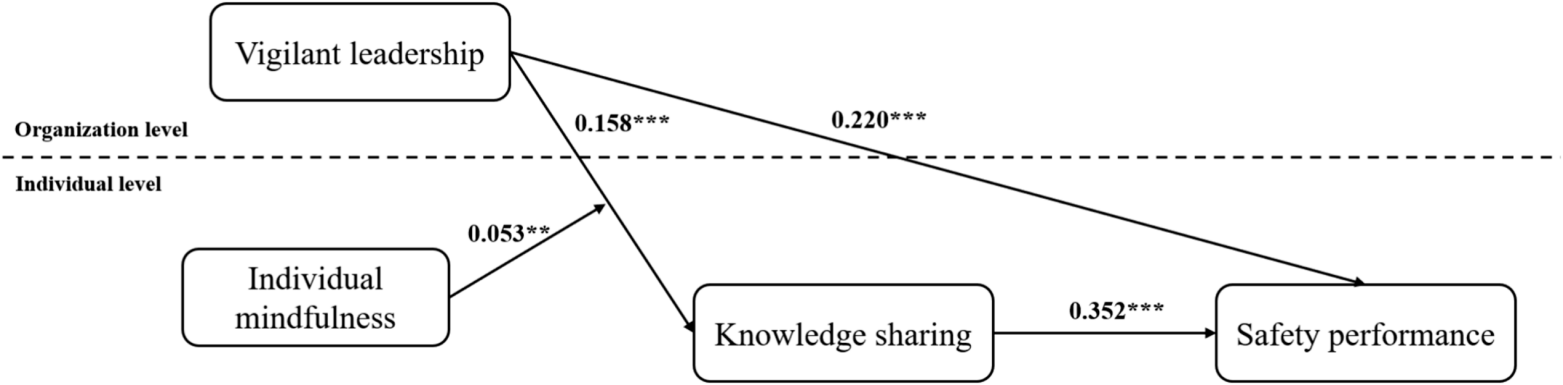
Path coefficients diagram of the model.

To provide a more intuitive exhibit of the core relationships about the research model, we added the path diagram with standardized coefficients, as shown in Figure 3.

## 5 Discussion

The present study introduces and validates a research model examining the roles of vigilant leadership, knowledge sharing, and individual mindfulness in enhancing safety performance within Chinese HSR organizations. Findings indicate that vigilant leadership not only has a direct, positive impact on the safety performance of HSR employees but also indirectly improves it through the stimulation of knowledge sharing. Moreover, individual mindfulness reinforces the positive relationship between vigilant leadership and knowledge sharing. The empirical evidence strongly supports all proposed hypotheses, with the research hypotheses aligning closely with the administrative assessment scores we have collected. Based on the empirical results, three theoretical contributions (5.1) and three practical implications (5.2) were identified. We also discuss the limitations and future directions (5.3).

### 5.1 Theoretical implications

First, by first introducing the vigilant leadership style to the context of Chinese HSR organizations, this study not only extends existing research on the determinants of safety performance and the potential influence of vigilant leadership but also enriches empirical studies on leadership within reliability-seeking organizations, thereby broadening the scope of occupational safety and health studies. Previous research mainly focused on the influence of general and safety-specific leadership styles, which are dominated by transformational leadership (43), transactional leadership (45), safety leadership (44), and safety-specific transformational leadership (100). Ethical leadership (101), authentic leadership (102), servant leadership (103), benevolent leadership (10–12), and empowering leadership (5) are also getting suitable attention. These leadership styles, however, are largely geared toward achieving short-term goals and employing positive methods or tactics to enhance employee safety. In Chinese HSR, where advocates “better safe than sorry,” vigilant leadership (future-focused and negative-oriented), which reflects this principle can be more conducive to safety and reliability. Specifically, vigilant leaders usually direct subordinates’ attention to potential problems and challenges, actively collaborate with experts, and implement “pour cold water” measures to address possible risks (104, 105). This stance not only boosts employees’ capacity to detect early warning signs but also aids in reducing the negative impacts of potential risks, ultimately contributing to the HSR’s safety and reliability and ensuring a high standard of driving performance. Our research also echoes Katz–Navon et al.’ (106) call for exploring the relations between safety behaviors within broader leadership categories.

Second, it extends the literature on leadership by examining how it influences safety performance by actively stimulating employees’ knowledge sharing from a novel theoretical perspective---information processing. Previous intermediary mechanisms acting on the safety performance of HSR employees mainly from job satisfaction (107), role breadth self-efficacy (108), organizational identification (109), employee resilience (110), work passion (52, 53), person-organization fit (111), and leader-member exchange (112), etc., our study confirmed that knowledge sharing is an irreplaceable mechanism that contributes to the improvement of HSR employees’ safety performance. In Chinese HSR organizations, before employees’ driving duties, leaders emphasize the conditions of today’s vehicles, the precautions and the possibility of encountering accidents or problems through group meetings. Employees attending night shifts also report the status of the lines and vehicles and share them with colleagues. These interactions highlight the critical intermediary role played by knowledge sharing in promoting employees’ safety performance. A large number of existing studies have examined the critical functions of knowledge sharing in organizations such as the coal mining industry (113), healthcare (114), and construction (6). HSR, however, does not pay much attention, once there is a data lag or asymmetry in knowledge information, the results can be huge and even life-threatening (9). We verified the significance of knowledge sharing in the HSR organization via empirical analysis. Therefore, this study not only enriches the intermediary mechanism between leadership and safety performance but also further adds to the knowledge sharing and organizational performance literature.

Third, our findings make a critical contribution by broadening the application scope of individual mindfulness. Although frequently treated as a moderating variable in other research contexts (35, 115), empirical evidence on individual mindfulness remains scarce within HSR organizations. Its vital role in HSR employees’ daily driving work has been proven by both working experience and the results of our data analysis. Our research addresses this gap by drawing on SIP theory to propose individual mindfulness as a critical moderator within HSR organizations. Specifically, this study shows that individual mindfulness, which reflects a self-awareness of processing the information changes in the external environment, strengthens the relationship between vigilant leadership and safety performance by focusing on the present moment. This highlights the essential role of personal traits in processing social information and leaders’ values, thus strengthening the discussion that individual mindfulness is critical in facilitating knowledge sharing in HSR employees. Moreover, this research also contributes to the SIP theory literature by enriching the individual’s way of constructing environmental information. The promotion of HSR employees’ knowledge sharing with a high level of mindfulness when processing cues from vigilant leaders demonstrates the constructive role played by individual mindfulness. Generally speaking, our findings affirm the crucial function that mindfulness plays within HSR organizations.

### 5.2 Practical implications

First, vigilant leadership is a crucial element in achieving safety and reliability in HSR organizations. Our empirical results demonstrate that vigilant leadership benefits HSR organizations because “Nip it in the bud” is a deeply embedded code of conduct within the industry’s culture. For this reason, the HSR organizations can identify leaders with heightened vigilance awareness by incorporating specific assessments into their recruitment processes. It is also feasible to identify and advance internal leaders who exhibit a vigilant attitude or to offer comprehensive training to potential ones, to cultivate vigilant leaders who align with the specific needs of the HSR organization. On the other hand, the achievement of employees’ safety performance cannot be realized without vigilance awareness. The driving adaptability test (a test requested by the State Railway Administration of China as a critical component in assessing the eligibility of candidates for employment as drivers) has been widely adopted in the Chinese HSR organization to ensure driving tasks. According to our findings, the National Railway Administration of China should integrate the cultivation of a vigilance mindset and behavior into the driving adaptability test, like designing targeted test questions to examine employees’ vigilance. Adding measures like utilizing VR technology in employees’ daily practical training to enhance their ability to handle on-the-spot challenges, effectively translating the proposition of vigilant leaders into actionable outcomes. This VR training can also be applied to leaders. In addition, eye tracker equipment can also be adopted to obtain the employee’s healthy condition and remind them when they suffer from a decrease in concentration and vigilance caused by consecutive driving tasks.

Second, managers in HSR organizations should provide a supportive, blame-free atmosphere (116) to encourage knowledge sharing and ensure psychological health. On the one hand, although many employees lack direct experience with major crises, such exposure invaluable for those novices. It is essential that experienced employees, particularly those with direct accident involvement, should share their insights and knowledge to foster a deeper understanding of these accidents and improve together. Moreover, by reviewing incident cases in routine meetings, managers can cultivate sustained operational vigilance among employees, thereby mitigating accident rates and fostering a positive psychological moods. On the other hand, the technology in HSR is advanced with time and constantly updated and iterative. Mastering the operation and utilization of diverse train types quickly is crucial for ensuring employees’ safety performance, and knowledge sharing is an effective approach. Managers in the HSR organization should continue to foster knowledge sharing among employees through both formal and informal channels (e.g., regular sharing by frontline employees or leaders in certain sessions). In addition, leaders can also reward constructive idea-sharing in various ways (117) to raise the whole organizational performance and safety.

Finally, HSR managers should attach great importance to cultivating employees’ mindfulness. Managers in the HSR organization should learn from general enterprises and institutions to adopt training practices about mindfulness. For instance, inviting experts to offer courses on Mindfulness Cognitive Therapy (MBCT) and integrating theory and practice are planned and designed in combination with the specific operation scenarios of HSR. Unlike conventional mindfulness intervention training courses, scenario vigilance exercises should be incorporated, and members are required to describe their psychological healthy conditions and thought processes when predicting risks. This kind of training course can be carried out quarterly, and can also be conducted on a daily basis. These approaches contribute significantly to maintaining a high standard of safety performance because maintaining a high level of mindfulness contributes to reducing employees’ rule-based, skill-based, and knowledge-based errors (34). On the other hand, employees with high mindfulness should be recruited or allocated resources to redesign existing employees’ thought patterns (118) and value their physical and mental health.

### 5.3 Limitations and future directions

Although the current study presents new findings and contributions, there are limitations. First, with the development of digital transformation in HSR organizations (119), some occupations show a disruptive development trend, and it is worth considering whether vigilant leadership still plays the same role in enhancing reliability. In other words, an examination about the vital role of digital transformation and AI for vigilance should be design and explored in the future research.

Second, this study explores individual mindfulness as a significant condition, other potential moderators could also be introduced, like individual difference variables (e.g., proactivity (120), and conscientiousness (121)) because both of them are critical to achieving organizational safety and reliability (122, 123). Moreover, whether the examined relationship holds for reliability-seeking teams—a common and dominant task-performing group in reliability-seeking organizations (124)—needs to be discussed in future research. The comparison with other high-reliability industries also should be noticed and test.

Third, the collectivist culture may enhance or weaken the effect of mindfulness on the main effect by using the sample from Chinese HSR organizations. Since individual mindfulness can be understood from both the Western perspective and the Eastern perspective (85, 125), the relationships presented in this study might show different results in individualism and collectivism. Therefore, it would be worthwhile to replicate the investigation of this study in different cultural contexts. Especially how the vigilant leadership can play a role in the HSR organizations across different cultures. Besides, the Chinese collectivist and hierarchical features may exert influence on the cultural specificity of Chinese HSR organizations. Hence, in future research, factors like cultural atmosphere (126) can also be considered as the boundary condition to further examine the hypothesis we proposed in this study, enhancing the external validity of the research findings.

Finally, research sample analysis revealed that 99.8% of employees and 100% of supervisors are men, which may influence the results of the hypothesis test. Future studies should expand the sample to include more female participants and incorporate diverse national backgrounds to further validate the hypotheses. Enhancing the transferability of the findings. Furthermore, as for the study design issues. On the one hand, this study adopted a time-lagged design, and by examining Harman’s single-factor test and a latent method factor to reduce the common method bias. However, we believe other methods, like vigilant leadership rated by supervisors, or using a marker variable, etc., could be used to further diminish the common method bias. It may be an interesting idea to further explore the hypotheses by using the leaders’ self-assessment questionnaire.

On the other hand, due to the influence of factors like the differences among raters, the units’ assessment situation, and the fluctuations in time periods, it is difficult to avoid potential rating biases. Therefore, future research should discuss the potential rating biases caused by the rater, unit, or time period. Even better, we hoped that future research could incorporate objective factual evidence, such as records of major events, accident statistics, or disciplinary archives, by employing Poisson/negative binomial models for supplementary validation. Thereby further enhances the validity and explanatory power of the conclusions drawn in this study.

Moreover, although the interaction effect in this study reached statistical significance within the current sample, its statistical power was relatively limited. Hence, we believe that future research can expand the sample size or across a broader range of research contexts to further validate the mechanisms of the interaction effects. Further, it is necessary to employ a multi-time point longitudinal design to conduct multilevel invariance testing by using data across multiple groups/waves, and strengthening the reliability of the causal interpretations in future research.

## 6 Conclusion

Based on the SIP theory, we proposed and empirically tested whether (direct effect), how (mediate effect), and when (moderate effect) vigilant leadership will influence HSR employees’ safety performance. There is a positive relationship between vigilant leadership and safety performance, which is transmitted by knowledge sharing. The individual mindfulness moderated the vigilant leadership and knowledge sharing relationship, such that the indirect effect is more positive when individual mindfulness is high than when it is low. We hope this study provides useful advancements for Chinese HSR organizations to enrich existing achievements in occupational health and safety of HSR employees.

## Data Availability

The data presented in this study are available on request from the corresponding author.

## References

[1] Roberts KH. Managing high reliability organizations. Calif Manag Rev. (1990) 32:101–13. doi: 10.2307/41166631

[2] Roberts KH. Some characteristics of one type of high reliability organization. Organ Sci. (1990) 1:160–76. doi: 10.1287/orsc.1.2.160, PMID: 19642375

[3] Kim YG. A quantitative accident analysis model on nuclear safety culture based on Bayesian network. Ann Nucl Energy. (2022) 166:108703. doi: 10.1016/j.anucene.2021.108703

[4] Gilmartin HM Hess E Mueller C Connelly B Plomondon ME Waldo SW . Learning environments, reliability enhancing work practices, employee engagement, and safety climate in VA cardiac catheterization laboratories. Health Serv Res. (2022) 57:385–91. doi: 10.1111/1475-6773.13907, PMID: 35297037 PMC8928023

[5] Mezentseva A Gracia FJ Silla I Martínez-Córcoles M. The role of empowering leadership, safety culture and safety climate in the prediction of mindful organizing in an air traffic management company. Saf Sci. (2023) 168:106321. doi: 10.1016/j.ssci.2023.106321, PMID: 41110882

[6] Sung WK Wang Y Wang L Luo X. Unraveling the drivers of construction safety knowledge sharing on online social media in engineering management. IEEE Trans Eng Manag. (2024) 71:14197–213. doi: 10.1109/TEM.2024.3430091

[7] Rosing F Boer D Buengeler C. Leader trait self-control and follower trust in high-reliability contexts: the mediating role of met expectations in firefighting. Group Org Manag. (2024) 49:10596011221104295:74–113. doi: 10.1177/10596011221104295

[8] Weaver B Kirk-Brown A Goodwin D Oxley J. Perceptions of psychosocial safety behaviour (PSB): qualitative insights on workplace psychosocial safety perceptions & actions within a policing context. Saf Sci. (2024) 172:106401. doi: 10.1016/j.ssci.2023.106401, PMID: 41110882

[9] Guo M Wei W Liao GL Chu FL. The impact of personality on driving safety among Chinese high-speed railway drivers. Accid Anal Prev. (2016) 92:9–14. doi: 10.1016/j.aap.2016.03.014, PMID: 27035394

[10] Wang AB Guo BY Yi ZW Fang WN. Research on enhanced situation awareness model with DMI visualization cues for high-speed train driving. Int J Hum Comp Interact. (2023) 40:6185–99. doi: 10.1080/10447318.2023.2247613

[11] Wang D Mao W Zhao C Wang F Hu Y. The cross-level effect of team safety-specific transformational leadership on workplace safety behavior: the serial mediating role of team safety climate and team safety motivation. J Saf Res. (2023) 87:285–96. doi: 10.1016/j.jsr.2023.05.001, PMID: 38081702

[12] Wang D Sun Z Zong Z Mao W Wang L Sun Y . The effect of benevolent leadership on safety behavior: a moderated mediation model. J Saf Res. (2023) 85:31–41. doi: 10.1016/j.jsr.2023.01.004, PMID: 37330881

[13] Wang ZT Li MK Zhang QD Wang Y Zhang W. High-speed train drivers' operation performance: key factors, models, and management implications. Int J Ind Ergon. (2023) 97:103482. doi: 10.1016/j.ergon.2023.103482

[14] Yan R Wu C Wang Y. Exploration and evaluation of individual difference to driving fatigue for high-speed railway: a parametric SVM model based on multidimensional visual cue. IET Intell Transp Syst. (2018) 12:504–12. doi: 10.1049/iet-its.2017.0289

[15] Grabowski M Martelli PF Roberts KH. Reliability-seeking virtual organizations at the margins of systems, resources and capacity. Saf Sci. (2023) 168:106327. doi: 10.1016/j.ssci.2023.106327, PMID: 41110882

[16] Hofmann DA Burke MJ Zohar D. 100 years of occupational safety research: from basic protections and work analysis to a multilevel view of workplace safety and risk. J Appl Psychol. (2017) 102:375–88. doi: 10.1037/apl0000114, PMID: 28125258

[17] Nielsen MB Skogstad A Matthiesen SB Einarsen S. The importance of a multidimensional and temporal design in research on leadership and workplace safety. Leadersh Q. (2016) 27:142–55. doi: 10.1016/j.leaqua.2015.08.003

[18] Hunt K Agarwal P Zhuang J. Monitoring misinformation on twitter during crisis events: a machine learning approach. Risk Anal. (2022) 42:1728–48. doi: 10.1111/risa.13634, PMID: 33190276

[19] Day GS Schoemaker PJ. Leading the vigilant organization. Strateg Leadersh. (2006) 34:4–10. doi: 10.1108/10878570610684784

[20] Zhao X. H. Liu Z. Q. (2018).” Research on precise marketing strategy of commercial Bank against the background of internet Finance.Advances in social science education and humanities research [proceedings of the 2018 2nd international conference on education, economics and management research (iceemr 2018)].” in 2nd international conference on education, economics and management research (ICEEMR), Singapore, Singapore.

[21] Day GS Schoemaker PJ. Are you a' vigilant leader'? MIT Sloan Manag Rev. (2008) 49:43. doi: 10.1109/EMR.2009.5235469

[22] Render ML Hirschhorn L. An irreplaceable safety culture. Crit Care Clin. (2005) 21:31–41. doi: 10.1016/j.ccc.2004.08.002, PMID: 15579351

[23] Weick KE Sutcliffe KM. Mindfulness and the quality of organizational attention. Organ Sci. (2006) 17:514–24. doi: 10.1287/orsc.1060.0196, PMID: 19642375

[24] Jiang Z Zhao XB Wang ZM Herbert K. Safety leadership: a bibliometric literature review and future research directions. J Bus Res. (2024) 172:114437. doi: 10.1016/j.jbusres.2023.114437

[25] Le Coze JC. Vive la diversite! High reliability organisation (HRO) and resilience engineering (RE). Saf Sci. (2019) 117:469–78. doi: 10.1016/j.ssci.2016.04.006

[26] Salancik GR Pfeffer J. A social information processing approach to job attitudes and task design. Adm Sci Q. (1978) 23:224–53. doi: 10.2307/2392563, PMID: 10307892

[27] Beus JM McCord MA Zohar D. Workplace safety: a review and research synthesis. Organ Psychol Rev. (2016) 6:352–81. doi: 10.1177/2041386615626243

[28] Christian MS Bradley JC Wallace JC Burke MJ. Workplace safety: a meta-analysis of the roles of person and situation factors. J Appl Psychol. (2009) 94:1103–27. doi: 10.1037/a0016172, PMID: 19702360

[29] Macrae C Draycott T. Delivering high reliability in maternity care: in situ simulation as a source of organisational resilience. Saf Sci. (2019) 117:490–500. doi: 10.1016/j.ssci.2016.10.019

[30] Henson JA Beehr T. Subordinates' core self-evaluations and performance predict leader-rated LMX. Leadersh Org Dev J. (2018) 39:150–68. doi: 10.1108/LODJ-06-2016-0162

[31] Paunova M. The emergence of individual and collective leadership in task groups: a matter of achievement and ascription. Leadersh Q. (2015) 26:935–57. doi: 10.1016/j.leaqua.2015.10.002

[32] Haavik TK Antonsen S Rosness R Hale A. HRO and RE: a pragmatic perspective. Saf Sci. (2019) 117:479–89. doi: 10.1016/j.ssci.2016.08.010

[33] Hassandoust F Johnston AC. Peering through the lens of high-reliability theory: a competencies driven security culture model of high-reliability organisations. Inf Syst J. (2023) 33:1212–38. doi: 10.1111/isj.12441

[34] Butler BS Gray PH. Reliability, mindfulness, and information systems. MIS Q. (2006) 30:211–24. doi: 10.2307/25148728

[35] Hülsheger UR van Gils S Walkowiak A. The regulating role of mindfulness in enacted workplace incivility: an experience sampling study. J Appl Psychol. (2021) 106:1250–65. doi: 10.1037/apl0000824, PMID: 32914993

[36] Burstein F Holsapple W van de Walle B Turoff M. Decision support for emergency situations. Handbook on. Decis Support Syst. (2008) 2:39–63. doi: 10.1007/s10257-008-0087-z

[37] Zhao C Gao Z Liu Y Fu Y. Watch out for icebergs: an investigation of vigilant leadership, antecedents, and consequences. Acad Manag Proc. (2018) 2018:13752. doi: 10.5465/AMBPP.2018.13752abstract

[38] Green JP Dalal RS Fyffe S Zaccaro SJ Putka DJ Wallace DM. An empirical taxonomy of leadership situations: development, validation, and implications for the science and practice of leadership. J Appl Psychol. (2023) 108:1515–39. doi: 10.1037/apl0001083, PMID: 37023297

[39] Griffin MA Neal A. Perceptions of safety at work: a framework for linking safety climate to safety performance, knowledge, and motivation. J Occup Health Psychol. (2000) 5:347–58. doi: 10.1037/1076-8998.5.3.347, PMID: 10912498

[40] Weiming G Qingren C Zhengquan X. The impact of ethical leadership on employee safety performance: the cross-level mediating effect of safety climate and psychological capital. Manag Rev. (2017) 29:116. doi: 10.14120/j.cnki.cn11-5057/f.2017.11.011

[41] Schulman PR. The negotiated order of organizational reliability. Adm Soc. (1993) 25:353–72. doi: 10.1177/009539979302500305

[42] Weick KE Sutcliffe KM Obstfeld D. Organizing and the process of sensemaking. Organ Sci. (2005) 16:409–21. doi: 10.1287/orsc.1050.0133, PMID: 19642375

[43] Nguyen VQ Turner N Barling J Axtell CM Davies S. Reconciling general transformational leadership and safety-specific transformational leadership: a paradox perspective. J Saf Res. (2023) 84:435–47. doi: 10.1016/j.jsr.2022.12.006, PMID: 36868673

[44] Clarke S. Safety leadership: a meta-analytic review of transformational and transactional leadership styles as antecedents of safety behaviours. J Occup Organ Psychol. (2013) 86:22–49. doi: 10.1111/j.2044-8325.2012.02064.x

[45] Wu X Qian Q Zhang M. Impact of supervisor leadership on construction worker safety behavior in China: the moderating role of social capital. Eng Constr Archit Manag. (2024) 31:1947–72. doi: 10.1108/ECAM-02-2022-0180

[46] Kark R Van Dijk D Vashdi DR. Motivated or demotivated to be creative: the role of self-regulatory focus in transformational and transactional leadership processes. Appl Psychol. (2018) 67:186–224. doi: 10.1111/apps.12122

[47] Kark R Van Dijk D. Keep your head in the clouds and your feet on the ground: a multifocal review of leadership–followership self-regulatory focus. Acad Manag Ann. (2019) 13:509–46. doi: 10.5465/annals.2017.0134

[48] Kaplan H. Event reporting, mindfulness and the high reliability organization: is the glass half empty? Vox Sang. (2002) 83:337–9. doi: 10.1111/j.1423-0410.2002.tb05330.x, PMID: 12617165

[49] Lau DC Liden RC. Antecedents of coworker trust: leaders' blessings. J Appl Psychol. (2008) 93:1130–8. doi: 10.1037/0021-9010.93.5.1130, PMID: 18808230

[50] Pan YF Li ZS Zhang ER Guo ZZ. A vigilance estimation method for high-speed rail drivers using physiological signals with a two-level fusion framework. Biomed Signal Process Control. (2023) 84:104831. doi: 10.1016/j.bspc.2023.104831

[51] Shaw TH Matthews G Warm JS Finomore VS Silverman L Costa PT. Individual differences in vigilance: personality, ability and states of stress. J Res Pers. (2010) 44:297–308. doi: 10.1016/j.jrp.2010.02.007

[52] Liu Y Wang Y Zhang F Liu S Liu P. Influence of team spiritual leadership on team green innovation performance from the perspective of social information processing. Curr Psychol. (2023) 42:25671–82. doi: 10.1007/s12144-022-03672-0

[53] Liu Y Zhang F Liu P Liu Y Liu S. “I’m energized to” & “I’m able to”: a dual-path model of the influence of workplace spirituality on high-speed railway drivers’ safety performance. Saf Sci. (2023) 159:106033. doi: 10.1016/j.ssci.2022.106033

[54] Chughtai MS Khan HS. Knowledge oriented leadership and employees’ innovative performance: a moderated mediation model. Curr Psychol. (2023):1–14. doi: 10.1007/s12144-023-04669-z, PMID: 37359696 PMC10132955

[55] Hodzic S Prem R Nielson C Kubicek B. When telework is a burden rather than a perk: the roles of knowledge sharing and supervisor social support in mitigating adverse effects of telework during the COVID-19 pandemic. Appl Psychol. (2023) 73:599–621. doi: 10.1111/apps.12491

[56] Grant RM. Toward a knowledge-based theory of the firm. Strateg Manag J. (1996) 17:109–22. doi: 10.1002/smj.4250171110

[57] Meng F Liu Y Zhang X Liu L. General knowledge-sharing and patient engagement in online health communities: an inverted U-shaped relationship. J Knowl Manag. (2023) 28:763–88. doi: 10.1108/JKM-12-2022-0986

[58] Devi NC. Paradoxical leadership and employee creativity: knowledge sharing and hiding as mediators. J Knowl Manag. (2023) 28:312–40. doi: 10.1108/JKM-10-2022-0779

[59] Erik Sveiby K. Disabling the context for knowledge work: the role of managers' behaviours. Manag Decis. (2007) 45:1636–55. doi: 10.1108/00251740710838004

[60] Nembhard IM Edmondson AC. Making it safe: the effects of leader inclusiveness and professional status on psychological safety and improvement efforts in health care teams. J Organ Behav. (2006) 27:941–66. doi: 10.1002/job.413

[61] Anand A. Shantakumar V. P. Muskat B. Singh S. K. Dumazert J.-P. Riahi Y. (2022). The role of knowledge management in the tourism sector: a synthesis and way forward. J Knowl Manag [Epub ahead of print].

[62] De Vries RE Van den Hooff B De Ridder JA. Explaining knowledge sharing: the role of team communication styles, job satisfaction, and performance beliefs. Commun Res. (2006) 33:115–35. doi: 10.1177/0093650205285366

[63] Berente N Lyytinen K Yoo Y King JL. Routines as shock absorbers during organizational transformation: integration, control, and NASA’S enterprise information system. Organ Sci. (2016) 27:551–72. doi: 10.1287/orsc.2016.1046, PMID: 19642375

[64] Vogus TJ Rerup C. Sweating the “small stuff”: high-reliability organizing as a foundation for sustained superior performance. Strateg Organ. (2018) 16:227–38. doi: 10.1177/1476127017739535

[65] Fauzi MA Nguyen M Malik A. Knowledge sharing and theory of planned behavior: a bibliometric analysis. J Knowl Manag. (2023) 28:293–311. doi: 10.1108/JKM-11-2022-0933

[66] Beck SJ Littlefield RS Weber AJ. Public meeting facilitation: a naïve theory analysis of crisis meeting interaction. Small Group Res. (2012) 43:211–35. doi: 10.1177/1046496411430531

[67] Cummings JN. Work groups, structural diversity, and knowledge sharing in a global organization. Manag Sci. (2004) 50:352–64. doi: 10.1287/mnsc.1030.0134, PMID: 19642375

[68] Fait M Scorrano P Mastroleo G Cillo V Scuotto V. A novel view on knowledge sharing in the Agri-food sector. J Knowl Manag. (2019) 23:953–74. doi: 10.1108/JKM-09-2018-0572

[69] Mumford EA Alfaro Hudak K Liotta MM O’Leary MS Ramey Sandra. Occupational prestige and job satisfaction in high-stress public safety work. Policing J Policy Pract. (2023) 17:paac049. doi: 10.1093/police/paac049

[70] Olan F Liu S Neaga I Alkhuraiji A. How knowledge sharing and business process contribute to organizational performance: using the fsQCA approach. J Bus Res. (2016) 69:5222–7. doi: 10.1016/j.jbusres.2016.04.116

[71] Labas A Courvisanos J. External business knowledge transmission: a conceptual framework. J Knowl Manag. (2022) 27:2034–57. doi: 10.1108/JKM-04-2022-0301

[72] Paoloni P Lombardi R Principale S. The impact of gender diversity on corporate social responsibility knowledge: empirical analysis in European context. J Knowl Manag. (2023) 27:2484–98. doi: 10.1108/JKM-07-2022-0512

[73] Langer E. Mindfulness isn’t much harder than mindlessness. Harv Bus Rev. (2016) 4:2023.

[74] Langer EJ Moldoveanu M. Mindfulness research and the future. J Soc Issues. (2000) 56:129–39. doi: 10.1111/0022-4537.00155

[75] Fraher AL Branicki LJ Grint K. Mindfulness in action: discovering how US navy seals build capacity for mindfulness in high-reliability organizations (HROs). Acad Manag Discov. (2017) 3:239–61. doi: 10.5465/amd.2014.0146

[76] Vogus TJ Rothman NB Sutcliffe KM Weick KE. The affective foundations of high-reliability organizing. J Organ Behav. (2014) 35:592–6. doi: 10.1002/job.1922

[77] Vendelø MT Rerup C. Collective mindfulness in a regenerating organization: ethnographic evidence from Roskilde festival. Saf Sci. (2020) 123:104537. doi: 10.1016/j.ssci.2019.104537

[78] McDonald N Callari TC Baranzini D Mattei F. A mindful governance model for ultra-safe organisations. Saf Sci. (2019) 120:753–63. doi: 10.1016/j.ssci.2019.07.031

[79] Sutcliffe KM Vogus TJ Dane E. Mindfulness in organizations: a cross-level review. Annu Rev Organ Psych Organ Behav. (2016) 3:55–81. doi: 10.1146/annurev-orgpsych-041015-062531

[80] Clarke DM. Managing the unexpected: resilient performance in an age of uncertainty (2nd edn) Karl E Weick and Kathleen M Sutcliffe (2007) Wiley & Sons, San Francisco; ISBN 978-0-7879-9649-9; HC; 194 pages; USD 27.05. J Manag Organ. (2008) 14:593–4. doi: 10.1017/S1833367200003072

[81] Saleem S Humayun S Raziq MM Iqbal MZ Ahmad M. Proactive personality and performance in the hospitality industry firms: mediating role of job crafting. Curr Psychol. (2023) 43:2516–33. doi: 10.1007/s12144-023-04356-z

[82] Wang X Li X Zhen F Zhang JH. How smart is your tourist attraction?: measuring tourist preferences of smart tourism attractions via a FCEM-AHP and IPA approach. Tour Manag. (2016) 54:309–20. doi: 10.1016/j.tourman.2015.12.003

[83] Brislin RW. Translation and content analysis of oral and written materials. In: Triandis HC Berry JW editors. Handbook of Cross-cultural Psychology. Boston: Allyn & Bacon, (1980) 2:389–444.

[84] Van Den Hooff B De Ridder JA. Knowledge sharing in context: the influence of organizational commitment, communication climate and CMC use on knowledge sharing. J Knowl Manag. (2004) 8:117–30. doi: 10.1108/13673270410567675

[85] Brown KW Ryan RM. The benefits of being present: mindfulness and its role in psychological well-being. J Pers Soc Psychol. (2003) 84:822–48. doi: 10.1037/0022-3514.84.4.822, PMID: 12703651

[86] Zheng J Gou X Griffin MA Goh YM Xia N. Temporal leadership, attentiveness, and safety behaviors: the moderating roles of abusive supervision and safety consciousness. Saf Sci. (2022) 147:105633. doi: 10.1016/j.ssci.2021.105633

[87] Menges JI Tussing DV Wihler A Grant AM. When job performance is all relative: how family motivation energizes effort and compensates for intrinsic motivation. Acad Manag J. (2017) 60:695–719. doi: 10.5465/amj.2014.0898

[88] Aiken LS West SG Reno RR. Multiple regression: Testing and interpreting interactions. United States, North America, Europe, South Asia and the Pan-Pacific region: SAGE Publication (1991) 14–22.

[89] Edwards JR Lambert LS. Methods for integrating moderation and mediation: a general analytical framework using moderated path analysis. Psychol Methods. (2007) 12:1–22. doi: 10.1037/1082-989X.12.1.1, PMID: 17402809

[90] Liu Y-y Liu P-q Liu D-x Liu S-z. Effect of paternalistic leadership on safety performance of transit bus drivers: activation effect of positive followership traits. Saf Sci. (2022) 153:105821. doi: 10.1016/j.ssci.2022.105821

[91] Bliese P. Within-group agreement, non-independence, and reliability: Implications for data aggregation and analysis. Multilevel theory, research, and methods in organizations Jossey-Bass (2000).

[92] Curcuruto M Renecle M Gracia F Morgan JI Tomas I. Improving workplace safety through mindful organizing: participative safety self-efficacy as a mediational link between collective mindfulness and employees’ safety citizenship. J Risk Res. (2024) 27:85–107. doi: 10.1080/13669877.2023.2293043

[93] Browne MW Du Toit SH. Automated fitting of nonstandard models. Multivar Behav Res. (1992) 27:269–300. doi: 10.1207/s15327906mbr2702_13, PMID: 26825725

[94] Marsh HW Hau K-T Grayson D. Goodness of fit in structural equation. In: Maydeu-Olivares A McArdle JJ editors. Contemporary psychometrics: A festschrift for Roderick P. McDonald. Lawrence Erlbaum Associates Publishers, (2002) 275–340.

[95] Nye CD. Reviewer resources: confirmatory factor analysis. Organ Res Methods. (2023) 26:608–28. doi: 10.1177/10944281221120541

[96] Gracia FJ Tomas I Martinez-Corcoles M Peiro JM. Empowering leadership, mindful organizing and safety performance in a nuclear power plant: a multilevel structural equation model. Saf Sci. (2020) 123:104542. doi: 10.1016/j.ssci.2019.104542

[97] Dyer NG Hanges PJ Hall RJ. Applying multilevel confirmatory factor analysis techniques to the study of leadership. Leadersh Q. (2005) 16:149–67. doi: 10.1016/j.leaqua.2004.09.009

[98] Fan P Ye L Yang S Song K Zhang H Guo M. High conflict, high performance? A time-lagged study on work-family conflict and family support congruence and safety performance. Saf Sci. (2024) 172:106403. doi: 10.1016/j.ssci.2023.106403

[99] Cohen J. Statistical power analysis for the behavioral sciences. New York: Routledge (2013).

[100] Wu YL Xu Q Jiang J Li Y Ji M You XQ. The influence of safety-specific transformational leadership on safety behavior among Chinese airline pilots: the role of harmonious safety passion and organizational identification. Saf Sci. (2023) 166:106254. doi: 10.1016/j.ssci.2023.106254

[101] Chughtai AA. Creating safer workplaces: the role of ethical leadership. Saf Sci. (2015) 73:92–8. doi: 10.1016/j.ssci.2014.11.016

[102] Sætrevik B Hystad SW. Situation awareness as a determinant for unsafe actions and subjective risk assessment on offshore attendant vessels. Saf Sci. (2017) 93:214–21. doi: 10.1016/j.ssci.2016.12.012

[103] Walumbwa FO Avolio BJ Gardner WL Wernsing TS Peterson SJ. Authentic leadership: development and validation of a theory-based measure. J Manag. (2008) 34:89–126. doi: 10.1177/0149206307308913

[104] Kumar M Rich N Kumar M Liu Y. Creating highly reliable health care organisations through reverse exchanges. Supply Chain Manag Int J. (2021) 26:371–84. doi: 10.1108/SCM-03-2020-0123

[105] Weick KE Sutcliffe KM. Managing the unexpected, vol. 9. San Francisco: Jossey-Bass (2001).

[106] Katz–Navon T Kark R Delegach M. Trapped in the middle: challenging the linear approach to the relationship between leadership and safety. Acad Manag Discov. (2020) 6:81–106. doi: 10.5465/amd.2017.0014

[107] Wei W Guo M Ye L Liao G Yang Z. Work-family conflict and safety participation of high-speed railway drivers: job satisfaction as a mediator. Accid Anal Prev. (2016) 95:97–103. doi: 10.1016/j.aap.2016.06.022, PMID: 27423429

[108] Chu F Liu S Guo M Zhang Q. I am the top talent: perceived overqualification, role breadth self-efficacy, and safety participation of high-speed railway operators in China. Saf Sci. (2021) 144:105476. doi: 10.1016/j.ssci.2021.105476

[109] Song K Guo M Chu F Yang S Xiang K. The influence of perceived human resource strength on safety performance among high-speed railway drivers: the role of organizational identification and psychological capital. J Saf Res. (2023) 85:339–47. doi: 10.1016/j.jsr.2023.04.001, PMID: 37330883

[110] Liu Y Liu S Liu R Liu Y. Leader mindfulness and employee safety behaviors in the workplace: a moderated mediation study. J Manag Psychol. (2024) 39:287–303. doi: 10.1108/JMP-03-2022-0128

[111] Liu S Chu F Guo M Liu Y. Authentic leadership, person-organization fit and collectivistic orientation: a moderated-mediated model of workplace safety. Leadersh Org Dev J. (2021) 42:1295–310. doi: 10.1108/LODJ-03-2020-0080

[112] Zhang N Liu S Pan B Guo M. Paternalistic leadership and safety participation of high-speed railway drivers in China: the mediating role of leader–member exchange. Front Psychol. (2021) 12:591670. doi: 10.3389/fpsyg.2021.591670, PMID: 34408689 PMC8366769

[113] Kirsch P Hine A Maybury T. A model for the implementation of industry-wide knowledge sharing to improve risk management practice. Saf Sci. (2015) 80:66–76. doi: 10.1016/j.ssci.2015.07.009

[114] Chang CW Huang HC Chiang CY Hsu CP Chang CC. Social capital and knowledge sharing: effects on patient safety. J Adv Nurs. (2012) 68:1793–803. doi: 10.1111/j.1365-2648.2011.05871.x, PMID: 22077142

[115] Vu TV Vo-Thanh T Chi H Nguyen NP Nguyen DV Zaman M. The role of perceived workplace safety practices and mindfulness in maintaining calm in employees during times of crisis. Hum Resour Manag. (2022) 61:315–33. doi: 10.1002/hrm.22101

[116] McFadden KL Henagan SC Gowen CR III. The patient safety chain: transformational leadership's effect on patient safety culture, initiatives, and outcomes. J Oper Manag. (2009) 27:390–404. doi: 10.1016/j.jom.2009.01.001

[117] Aanestad M Jensen TB. Collective mindfulness in post-implementation IS adaptation processes. Inf Organ. (2016) 26:13–27. doi: 10.1016/j.infoandorg.2016.02.001

[118] Fiol CM O'Connor EJ. Waking up! Mindfulness in the face of bandwagons. Acad Manag Rev. (2003) 28:54–70. doi: 10.5465/amr.2003.8925227

[119] Garg P Gupta B Sar A Graham G Shore AP. Development and validation of an instrument to measure the perceived benefits of digitalization in manufacturing. IEEE Trans Eng Manag. (2024) 71:8288–306. doi: 10.1109/TEM.2024.3390434

[120] Zhu Y Quansah PE Obeng AF Minyu G. High-performance work systems and safety performance in the mining sector: exploring the mediating influence of workforce agility and moderating effect of safety locus of control. Curr Psychol. (2023) 42:25100–26. doi: 10.1007/s12144-022-03606-w

[121] Christian MS Garza AS Slaughter JE. Work engagement: a quantitative review and test of its relations with task and contextual performance. Pers Psychol. (2011) 64:89–136. doi: 10.1111/j.1744-6570.2010.01203.x

[122] Gajda D Zbierowski P. Exploring the consequences of mindfulness at work: the impact of mindful organizing on employee attitudes and behavior toward work and organization. Pers Rev. (2023) 52:2342–62. doi: 10.1108/PR-05-2020-0385

[123] Harolds JA. Quality and safety in healthcare, part LXXXVI teamwork and the comprehensive unit-based safety program. Clin Nucl Med. (2023) 48:E101–3. doi: 10.1097/RLU.0000000000003587, PMID: 33782289

[124] Williams G Irving G Wright AL Middleton S. Managing job-related diversity processes in high-reliability teams in the emergency department. Br J Manag. (2022) 33:502–18. doi: 10.1111/1467-8551.12450

[125] Vogus TJ Sutcliffe KM. Organizational mindfulness and mindful organizing: a reconciliation and path forward. Acad Manag Learn Educ. (2012) 11:722–35. doi: 10.5465/amle.2011.0002c

[126] Amabile TM Conti R Coon H Lazenby J Herron M. Assessing the work environment for creativity. Acad Manag J. (1996) 39:1154–84. doi: 10.2307/256995

